# Characterisation of inflammatory processes in *Helicobacter pylori*-induced gastric lymphomagenesis in a mouse model

**DOI:** 10.18632/oncotarget.5948

**Published:** 2015-10-01

**Authors:** Pauline Floch, Amandine Marine Laur, Victoria Korolik, Delphine Chrisment, David Cappellen, Yamina Idrissi, Pierre Dubus, Francis Mégraud, Philippe Lehours

**Affiliations:** ^1^ University of Bordeaux, Bacteriology Laboratory, F-33000 Bordeaux, France; ^2^ Inserm U853, F-33000 Bordeaux, France; ^3^ Institute for Glycomics, Griffith University, Gold Coast, QLD, Australia; ^4^ University of Bordeaux, EA 2406, F-33000 Bordeaux, France

**Keywords:** cytokines, chemokines, MALT lymphoma, TNF, animal model

## Abstract

Gastric MALT lymphoma (GML) can be induced by *Helicobacter pylori* infection in BALB/c mice thymectomised at day 3 post-birth (d3Tx). This represented a unique opportunity to investigate the inflammatory process involved in the recruitment, proliferation and structuration of lymphoid infiltrates in the gastric mucosa of mice developing GML. Complementary molecular and proteomic approaches demonstrated that Th1 and Th2 cytokines were upregulated, along with activators/regulators of the lymphoid response and numerous chemokines. Interleukin-4, interferon γ, lymphotoxin-α and -β were significantly upregulated and correlated with the inflammatory scores for all the d3Tx mice. GML lesions in d3Tx mice infected with *H. pylori* were associated with the presence of the inflammatory response. The dysregulation of numerous members of the tumour necrosis factor superfamily was also evident and suggests that they could play an important role in GML pathology, especially in light of their ability to promote and control lymphocyte proliferation.

## INTRODUCTION

*Helicobacter pylori* is a helical rod-shaped Gram-negative bacterium that colonises the human gastric mucosa with colonisation rates of about 50% worldwide. The infection causes inflammation (gastritis) which is first superficial and asymptomatic but may evolve, under the influence of virulence factors, towards other pathologies such as gastric or duodenal ulcer, gastric adenocarcinoma or gastric mucosa associated lymphoid tissue (MALT) lymphoma (GML) [1] [2] [3].

GML is a rare consequence of chronic inflammation in the gastric mucosa caused by *H. pylori* infection [4]. This pathology is characterised by organised lymphoid infiltrates (similar to intestinal Peyer's patches), mainly constituted of neoplastic B cells from the marginal zone. Chronic inflammatory diseases caused by pathogens or even autoimmune-induced processes are often associated with the neogenesis of lymphoid tissues in non-lymphoid organs such as the stomach [5] [6].

Several studies have clearly demonstrated that human GML biopsies are infiltrated by large numbers of T-helper cells expressing interleukin-4 (IL-4) and other T-helper cell type 2 cytokines. Intratumoural T cells sustain tumour B cell proliferation in a CD40/CD40L dependent manner [7] [8] [2] [9]. As a general rule, the tendency of a response to become polarized toward Th1 or Th2 effectors is influenced by a combination of host genetic factors and the type and amount of antigen that is encountered. Chemokines also play well defined roles in the initiation of T cell immune responses and homing of effector T cells to sites of inflammation [10]. Furthermore, chemokines are the principal regulators of lymphocyte and dendritic cell migration, lymphoid organ development, and lymphoid homeostasis [10] [6]. The investigation of the gastric inflammatory response at a GML stage was therefore of major importance in order to better characterise the driving forces that could favour the emergence of lymphoid infiltrates in an organ that is naturally devoid of lymphoid tissue.

Here we have used the material previously obtained using BALB/c mice in which we showed that *H. pylori* infection can induce the emergence of GML lesions 12 month post-infection in thymectomised 3 days after birth (d3Tx) only, but not in non-thymectomised (NTx) infected mice [11].

The main goal of the present study was to compare inflammatory responses of GML model d3Tx mice and NTx mice. An extensive investigation of the pro-inflammatory local response in animals presenting a lymphoid infiltration was conducted in order to obtain more information on the effectors stimulating B cell expansion. First, the expression of inflammatory molecules was evaluated by a global approach using a qRT-PCR array on selected samples to identify dysregulation of relevant genes. Then the gastric overexpression of some identified target genes was confirmed on a larger number of samples. To verify these data, the inflammatory response was investigated by protein array. Our results indicate that GML-developing mice developed a local inflammatory response that is likely to trigger the recruitment of leukocytes and promote lymphocyte proliferation and the emergence of lymphoid structures. We postulate that tumour necrosis factor superfamily members may play a pivotal role in the emergence and proliferation of lymphoma cells.

## RESULTS

### Investigation of the gastric inflammatory response by PCR array

To establish a baseline, the gastric inflammatory responses in the 3 non-infected (NI) non-thymectomised (NTx) and the 3 NI thymectomised (d3Tx) mice were first compared. There was no significant upregulated or downregulated target genes among the two groups (data not shown). Therefore the Ct values of these 6 NI were considered as a single control group and used for identification of the dysregulated expression of chemokines and cytokines in each infected group (NTx and d3Tx). The distribution of dysregulation values higher or lower than cut-off of 2 are shown in Table 1.

**Table 1:** Upregulated targets in thymectomised and non-thymectomised infected mice

Target	Symbol	d3Tx	NTx
Chemokine (C-C motif) ligand 1	CCL1	**3.3776**	1.1702
Chemokine (C-C motif) ligand 12	CCL12	**4.3967**	**3.2908**
Chemokine (C-C motif) ligand 2	CCL2	**4.8391**	1.6392
Chemokine (C-C motif) ligand 20	CCL20	**2.1021**	**5.7411**
Chemokine (C-C motif) ligand 3	CCL3	**12.0991**	1.3768
Chemokine (C-C motif) ligand 4	CCL4	**15.1562**	1.3756
Chemokine (C-C motif) ligand 5	CCL5	**9.0041**	−1.3194
CD40 ligand	CD40L	**2.997**	1.3188
Chemokine (C-X-C motif) ligand 10	CXCL10	**2.213**	−1.4186
Chemokine (C-X-C motif) ligand 11	CXCL11	**2.4126**	1.2647
Chemokine (C-X-C motif) ligand 13	CXCL13	**2.8116**	1.5674
Chemokine (C-X-C motif) ligand 9	CXCL9	**8.9471**	1.5075
Fas ligand (TNF superfamily, member 6)	FasL	**2.5457**	1.178
Interferon gamma	INFγ	**6.0846**	1.4778
Interleukin 12B	IL-12b	**4.2728**	1.3981
Interleukin 13	IL-13	**3.6621**	1.3101
Interleukin 16	IL-16	**3.4635**	1.4876
Interleukin 1 alpha	IL-1a	**2.323**	1.3689
Interleukin 1 beta	IL-1b	**3.2729**	1.5132
Interleukin 1 receptor antagonist	IL-1rn	**4.9337**	1.4163
Interleukin 4	IL-4	**4.9508**	1.6454
Interleukin 6	IL-6	**3.0502**	1.1797
Lymphotoxin B	Ltb	**6.726**	1.7984
Tumour necrosis factor	TNF	**3.6536**	1.4453
Tumour necrosis factor (ligand) superfamily, member 10 (TRAIL)	TNFsf10	**4.6554**	−1.16
Tumour necrosis factor (ligand) superfamily, member 11 (TRANCE/RANKL)	TNFsf11	**3.8508**	1.2266
Tumour necrosis factor (ligand) superfamily, member 13b (BAFF)	TNFsf13b	**2.3759**	1.567
Chemokine (C motif) ligand 1	XCL1	**5.4538**	**2.1276**

For each group 8 mice were included and fold-regulation values were compared to 6 non-infected animals. Those higher or lower than 2 are highlighted in bold for both infected groups of mice. The complete designation of each target is indicated as well as the common symbol. d3Tx: thymectomised infected mice; NTx: non-thymectomised infected mice.

A total of 3 chemokine encoding genes (CCL12, CCL20 and Xcl1) were found upregulated in infected NTx mice when compared to the NI control group. In contrast, a total of 25 genes were found specifically upregulated in infected d3Tx mice (Table 1): 1) Th1 associated cytokines such as IFNγ, IL-12, and Th2 associated cytokines such as IL-4, IL-6 and IL-13, as well as the lymphocyte chemoattractant factor IL-16; 2) activators or regulators of the lymphocytic response such as co-stimulation signaling molecule CD40L, LTβ, IL-1 (IL-1α, IL-1β and IL-1 receptor antagonist), FasL and other members of the TNF-superfamily such as TNF, TRAIL, RANKL and BAFF; and 3) numerous chemokines of CCL (CCL1, 2; 3, 4, 5) or CXCL type (9, 10, 11, 13). The presence of all of these cytokines/chemokines is in agreement with a mixed Th1/Th2 response and with the recruitment and sustainment of a lymphocytic proliferation. Neither Th17 (such as IL-17, IL-21, IL-23) cytokines, nor regulatory T cells (Tregs) (IL-10 or TGFβ) targets were upregulated in infected animals. In conclusion, mice developing GML exhibited an inflammatory response that could trigger leukocyte recruitment and B lymphocyte proliferation. The inflammatory background appeared Th1 and Th2 polarized.

### Relative expression levels of IL-4 and IFNγ cytokines and lymphotoxins in NTx and d3Tx mice

**Figure 1:**
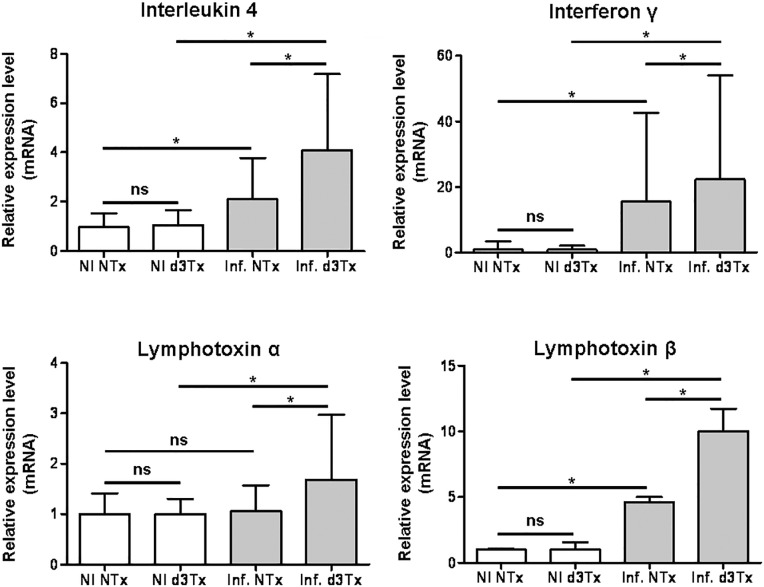
Relative expression levels of IL-4, IFNγ and lymphotoxins α and β in infected NTx and d3Tx mouse stomachs in comparison with expression levels in NI mice Expression levels for infected NTx (*n* = 40) and d3Tx (*n* = 29) mouse groups were normalised in comparison with respective NI NTx (*n* = 10) and NI d3Tx (*n* = 8) control group expression levels. Data are plotted as bar graphs displaying the average/mean ± standard deviation for each group, *p < 0.05, ns = not significant. *Gusb* was used as housekeeping gene. Similar results were obtained with *HPRT* (not shown).

A set of the upregulated targets identified above were investigated further using a larger number of samples in order to evaluate more precisely their specific dysregulation at the lymphoma stage. The relative expression level of IL-4 (associated with a Th2 immune response) in stomachs of infected NTx mice was found to be approximately twice that of NI NTx mice (*p < 0.05). In infected d3Tx mouse stomachs, it was 4 times higher than in the NI d3Tx control group (*p < 0.05), and approximately 2 times higher in infected d3Tx mice in comparison with infected NTx mice (*p < 0.05) (Figure 1).

The same phenomenon was observed for INFγ (associated with a Th1 immune response) with relative expression levels 15 and 22 times superior to controls in infected NTx and d3Tx mice, respectively (*p < 0.05). The expression level was again higher in infected d3Tx mice in comparison with infected NTx (*p < 0.05).

Among the TNFα superfamily members, LTα and LTβ were both investigated even though LTβ only was upregulated in the PCR array. Indeed it has been suggested that these lymphotoxins, working together, represent key molecules in the development of lymphoid structures in general [12] [13] and in the stomach specifically [14]. It is noteworthy that the expression levels of LTα were unchanged in infected NTx mice in comparison with the NI controls, but were significantly elevated in infected d3Tx mice (*p < 0.05). The expression levels of LTβ in infected NTx mice were 4 to 5 times higher to that of the NI control (*p < 0.05), and 10 times higher to that of the infected d3Tx control (*p < 0.05). The relative level of LTβ in infected d3Tx mice was approximately twice that of the infected NTx group (*p < 0.05) (Figure 1).

**Figure 2:**
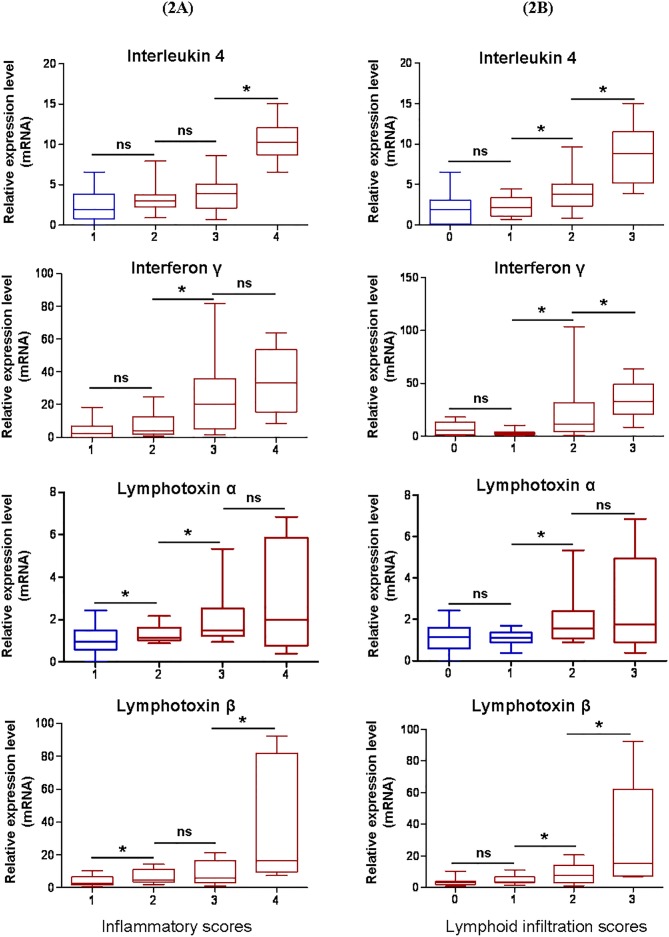
Correlation between relative expression levels of IL-4, IFNγ and lymphotoxins α and β and histological scores of infected d3Tx mice Relative expression levels for each gene of interest were classified according to histological scores obtained for each infected d3Tx mouse. **A.** Correlation of relative expression levels in comparison with inflammation scores (*n* = 8, 9, 9 and 3 respectively for scores of 1, 2, 3 and 4). **B.** Correlation of relative expression levels in comparison to lymphoid infiltrates scores (*n* = 5, 8, 12 and 4 respectively for scores of 0, 1, 2 and 3). Graphic representations as box plots, with the box representing 50% of values around the median (horizontal line) and the whiskers representing the minimum and maximum of all the data, *p < 0.05, ns = not significant. In blue not significant expression levels when compared to the NI d3Tx control group, in red significant expression levels when compared to this same group (*p* < 0.05). *Gusb* was used as housekeeping gene. Similar results were obtained with *HPRT* (not shown).

These relative expression levels obtained for each infected d3Tx mouse were classified according to histological scores of inflammation and lymphoid infiltrates as described previously [11]. In general, a positive correlation was observed with the scores for all cytokines/LTs investigated (Figures 2A and 2B). In comparison with the NI d3Tx control group, the 4 targets were all significantly overexpressed in mice with inflammation and lymphoid infiltrate scores of 2 or higher which correlated with mice with GML lesions.

Hence, infected d3Tx mice seemed to develop a more pronounced Th2 (IL-4) and Th1 (IFNγ) response than infected NTx mice. Overexpression of the 2 active subunits of LT only appears in d3Tx mice and correlated with the structuring of lymphoid follicles in their stomachs.

### Analysis of the gastric inflammatory response by cytokine antibody array

Overexpression of genes encoding cytokines and chemokines was verified at the protein level by cytokine antibody arrays. Inflammatory response in 4 infected versus 3 NI d3Tx mice (as described in material and methods) was therefore compared to the relative abundance of cytokines and chemokines in the stomach.

The fold-regulation values equal to or higher than 2 are shown in Supplemental Table S1. A total of 65 proteins among the 144 examined, were overproduced in infected d3Tx mice: 1) Th1 cytokines such as IL-2 and its receptor IL-2 Rα, IL-12A and IL-12B, INFγ; Th2 cytokines such as IL-13 and Th17 cytokines such as IL-17 and IL-21; 2) numerous activators or regulators of the lymphocytic response such as co-stimulation signal CD40L and its receptor CD40, IL-1 (IL-1α, IL-1β), IL-3 and its receptor IL-3 Rβ, IL-20, IL-28, TNFα and its receptors (sTNF RI and RII) and other members of the TNF-superfamily such as GITR, TWEAK, CD27, CD30 (and their respective ligands) and TACI; 3) numerous chemokines of CCL- (1, 3, 5, 11, 17, 19, 20, 25, 27) or CXCL-type (1, 2, 5, 10, 12, 13, 16); 4), as well as other proteins (Supplemental Table S1). Production of these cytokines and chemokines correlates with a mixed Th1/Th2/Th17 inflammatory response and with the recruitment and sustainment of a lymphocytic proliferation.

**Figure 3:**
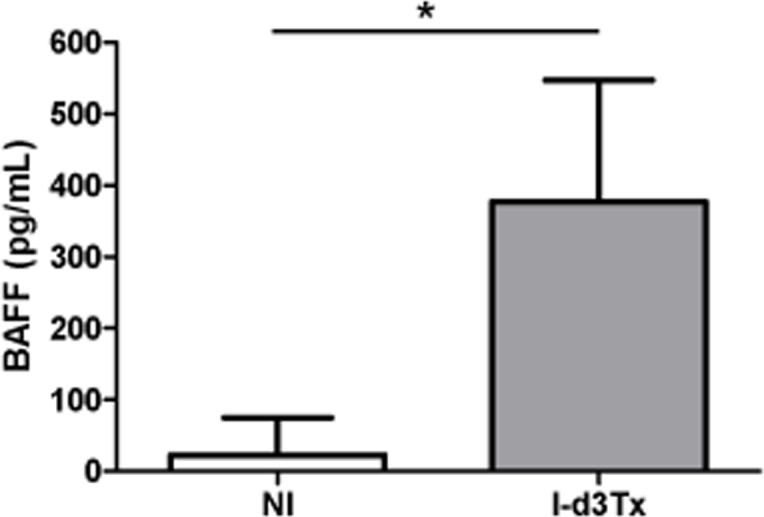
Determination of BAFF overproduction in gastric biopsies from thymectomised mice BAFF levels for non-infected d3Tx (NI, *n* = 5) and infected d3Tx (I-d3Tx, *n* = 6) mouse groups were determined by ELISA. Each sample was tested in duplicate. Data are plotted as bar graphs displaying the average/mean ± standard deviation for each group, *p < 0.05, ns = not significant.

Among the 28 targets identified at the transcriptional level in infected d3Tx mice, 13 (46.4%) were also found to be over-produced in the cytokine antibody array (Table 2). Over-production of twelve target genes that were not detected at the transcriptional level (likely due to high turnover of mRNA) were detected by cytokine antibody array (Table 2). Presence of cytokines such as Th17, IL-17 and IL-21, suggests a role for Th17 response in the inflammatory process leading to development of GML. Of the 4 targets that were found over-expressed by PCR array, but not determined by cytokine array, BAFF was investigated further. We confirmed by ELISA quantification that it was over-produced (around 16.6 times higher in infected d3Tx mice compared to NI controls) in mice developing GML (Figure 3). Moreover, the cytokine antibody array allowed the identification of 22 targets that were not included in the PCR array panel and, among these, numerous members of TNF-superfamily (Table 2). Considering the respective pro-inflammatory, proliferative and/or pro-apoptotic activities of these latter targets, it is likely that they may play an important role in the GML microenvironment. Finally, Tregs (IL-10 or TGFβ) cytokines were never found to be upregulated in infected animals in our analyses.

**Table 2:** Targets upregulated at transcriptional and translational levels in infected thymectomised mice

	A	B	C	D	E
Chemokines	CCL (1, 3, 5, 20) CXCL (10, 13)	CCL (11, 17, 19) CXCL (1, 5, 12, 16)	CCL (2, 12) CXCL (9, 11) Xcl1	CCL (25, 27) CXCL2	CCL4
Th1 cytokines	IFN-γ IL-12B	IL-2 IL-12A		IL-2 Rα	
Th2 cytokines	IL-13		IL-4 IL-6	IL-6 R	
Th17 cytokines		IL-17 IL-21			
Lymphocyte activation and regulation factors	CD40 L IL-1α IL-1β TNFα	IL-3	FasL IL-1 RA TRANCE	CD27 (+L) CD30 (+L) CD40 Fcg RIIB GITR (+L) GAS 6 IL-3 Rβ IL-20 IL-28 sTNF RI sTNF RII TACI TWEAK(+L)	IL-16 LTβ TRAIL BAFF
Number of targets	(13)	(12)	(10)	(22)	(5)

A: over-expressed targets by PCR and cytokine antibody array; B: over-produced targets by cytokine antibody array only; C: over-expressed targets by PCR array only; D: over-produced targets by cytokine antibody array (not determined by PCR-array), E: over-expressed targets by PCR array (not determined by cytokine antibody array). Members of the TNF-superfamily are underlined. (cytokine antibody array and PCR array : fold change > or =2).

In conclusion, antibody array results confirmed and complemented those obtained by qRT-PCR array and highlighted the putative involvement of members of the TNF-superfamily in the inflammatory process.

## DISCUSSION

This study focused on the gastric inflammatory response involved in the emergence of GML. By using the gastric biopsies obtained in our previous study [11] from *H. pylori*-infected d3Tx mice that developed GML, we showed the upregulation at transcriptional and translational levels of numerous gene targets which, when considered together, highlight the molecular processes likely to trigger emergence of lymphoid structures in the gastric mucosa.

Three genes were found to be dysregulated in both NTx and d3Tx models, a common signature of *H. pylori* infection in BALB/c mice. Xcl1, a T cell attractant factor [15], CCL20, implicated in the formation and function of mucosal lymphoid tissues via chemoattraction of lymphocytes (including regulatory T cells) and dendritic cells [16], and CCL12, involved in the recruitment of eosinophils, monocytes and lymphocytes [17] were all upregulated in infected NTx and d3Tx animals. This common inflammatory trait could initiate the recruitment of leukocytes at the site of infection in both models. However, the inflammatory response within the structuration of lymphoid infiltrates was highly evident in infected d3Tx mice only [11]. Considering the role of the target genes that we found to be specifically upregulated in d3Tx mice developing GML, several mechanisms of how GML is controlled or favoured could be hypothesised.

Some of the inflammatory factors identified in this study could favour the recruitment of leukocytes and promote lymphocyte proliferation in mice developing GML. Th1 (IFNγ) and Th2 (IL-4 and IL-13) mixed inflammatory response was indeed observed in both of our NTx and d3Tx mouse models. IFNγ and IL-4 transcript levels were significantly higher in the d3Tx model and the increase in expression of these pro-inflammatory factors correlates with the increase of the histological scores of inflammation and lymphoid infiltrates. Our results are in agreement with previous data found in GML patients, in which tumour infiltrating T cells were comprised of a heterogenous population consisting of multiple functional subsets which secreted different combinations of both cytokines, Th1- and Th2-type [18].

Cytokines, in particular IL-12 and IL-4, are dominant factors that regulate T cell differentiation. However, alternative pathways for Th1 and Th2 polarization exist [10]. For example, T cell-derived chemokines might influence T cell priming and differentiation and are likely to be especially important at sites of inflammation.

CCL2 is considered to be a Th2-inducing chemokine via two major pathways: either the reduction of IL-12 production by antigen presenting cells or the enhancement of IL-4 production by activated T cells. Aside from CCL2 activity, it has been shown that, when high amounts of CCL3 or CCL5 are present at a site of T cell activation, a cell-mediated Th1-type response is favoured [19] [10]. Finally, CCL19 may play a role in dampening an inflammatory Th1-type response [10].

TNFα is a very strong stimulus for chemokine expression. It has been shown specifically that that TNFα synergized with IL-4, and to a lesser extent with IFNγ and IL-13, to release chemokines such as CCL1 or CXCL1. [20] There probably is a local crosstalk between all of these partners that induces and sustains the inflammatory response. While T cell cytokines may, in the end, be driving this activation, the role of the early innate response and macrophage/dendritic cell/epithelial cell activation in recruiting the T cells to the gastric tissue has to be investigated further. The study of the inflammatory response at earlier time points after infection could be very interesting to determine if the early events are different in NTx and d3Tx mice.

A range of factors has been related to the structuration of lymphoid infiltrates in gastric mucosa. CCL1 has been implicated in leukaemia and lymphoma, in particular via an autocrine loop that protects cells from apoptosis [21]. It also promotes migration of tumour cells towards lymph nodes [22]. LTs, CXCL13 and CCL19 have been shown to influence the organisation and function of tertiary lymphoid structures and maintenance of lymphoid tissue microarchitecture [5] [6].

Similarly, LT-mediated (TNF-superfamily) signalling has been shown to actively contribute to effector immune responses and is present in different heterotrimeric (LTα1β2 or LTα2β1) or soluble homotrimeric (LT_α3_) forms. It is interesting to note that in the present study, only infected d3Tx mice overexpressed both LTα and LTβ subunits, both necessary for its biological action. This overexpression was also correlated to inflammation and lymphoid infiltrate scoring. Lack of significant lymphoid infiltrates in NTx mouse stomachs is therefore consistent with previous observation that a deletion of LTα leads to a defect in lymphoid organ development [23]. Moreover, LT might also be essential for Th2 cell development, similar to that shown for helminth infection [24]. Even more interesting is the evidence of a positive feedback loop between CXCL13 and the upregulation of LTα1β2 on B cells for the maintenance of B cell follicles which has been suggested previously [5] [6]. Migration of lymphocytes into the gastric mucosa and structuration of lymphoid infiltrates in GML could therefore be influenced by presence of LT. These results are in line with previous reports, where CXCL13 was shown to be crucial for lymphoid infiltrate formation in GML [25] in other animal models [26].

Finally, it has been shown that in GML CD40 and its receptor CD40L, play an important role in the co-stimulation of B cells [8]. CD40 signalling in B cells is also known to promote germinal centre formation, and to be essential for the survival of many cell types including gastric cancer B cells under normal and inflammatory conditions [27]. Therefore, the upregulation of these factors could favour the emergence of lymphoid structures in GML.

TNF-superfamily members could play important roles in GML pathogenesis. Interestingly our study also revealed upregulation of numerous members of the TNF-superfamily, suggesting an important role of this family in the GML microenvironment. All members of the TNF-superfamily, without exception, exhibit pro-inflammatory activity, in part via activation of the transcription factor NF-κB. This ability to activate a pro-inflammatory transcription factor provides members of the TNF-superfamily with carcinogenic activities by regulating the expression of genes linked to tumour cell survival, proliferation, invasion, angiogenesis and metastasis [28].

**Figure 4:**
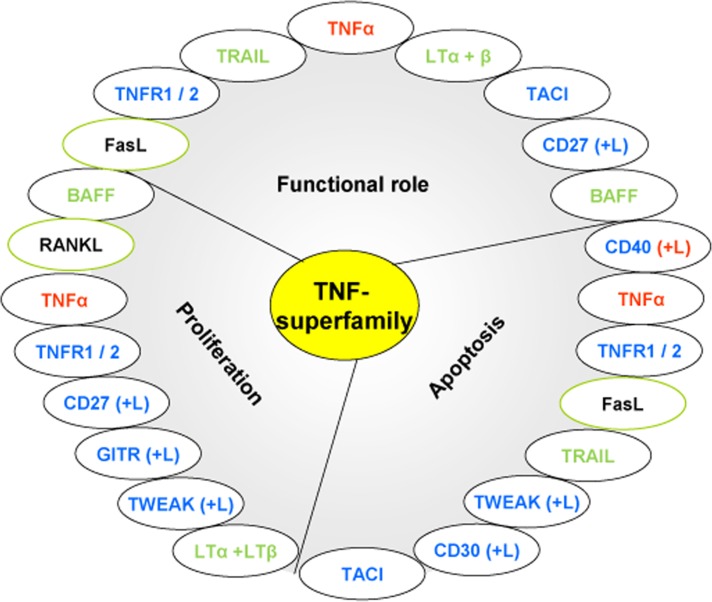
Roles of the upregulated members of the TNF-superfamily in our study Each member of the TNF-superfamily can exhibit various functions. (In red: members upregulated at molecular and protein levels; in blue: members overproduced at the protein level (mRNA expression not determined); in green: members overexpressed at molecular level (protein production not determined); in black framed in green: members overexpressed at transcriptional level but not at protein level).

Several members such as BAFF, CD27, GITR, LTα and LTβ, and RANKL which are upregulated in mice developing GML (either at the transcriptional or translational level), exhibit proliferation activation of haematopoietic cells, in part by activating various mitogen-activated kinases. In contrast, other members of this family such as CD40, FasL, TRAIL and CD30, play a role in apoptosis. TRAIL can regulate lymphocyte functions in the periphery by inhibiting T cells cell-cycle progression [29]. Other members such as TNFα, FasL and TWEAK display both pro-apoptotic and proliferative activities (Figure 4). In some cell types, TWEAK functions not as a death factor but as a cell survival [30] or proliferation factor [31]. Therefore, most members of the TNF-superfamily have both beneficial and potentially harmful effects [32] and a disbalance between their respective proliferative and/or pro-apoptotic activities would create a favourable environment for GML development.

Specific attention needs to be directed to BAFF, a member of the TNF-superfamily, as it appears to play a critical role in GML development and critical to B-cell survival and maturation [33] [34]. BAFF expression is known to be regulated in response to cytokines such as INFγ and CD40L that we also found to be upregulated in GML-developing mice. Numerous studies have demonstrated that BAFF-signalling induces expression of anti-apoptotic molecules such as Bcl-2 in B cells, confirming its significant role in promoting B-cell survival [35] [36]. Furthermore, BAFF plays a role in lymphocyte differentiation as B-cell differentiation is completely abolished in BAFF-deficient mice [37]. Aberrant BAFF expression was reported to be associated with *H. pylori* independant growth of GML [38]. Here were propose that dysregulation of BAFF could play a major role in stimulation of B cell proliferation even in *H. pylori* GML positive cases.

BAFF is also able to drive Th17 response during *H. pylori* infection; the Th17 response that was characterized in our model involves upregulation of IL-17 and IL-21 at the protein level [39]. Interestingly, it has been shown that IL-21 has a role in sustaining both Th1 and Th17 effector cell responses [40]. It was also shown that IL-21 contributes to *H. pylori* specific antibody responses, suggesting this cytokine may have an effect on B cell proliferation [40].

It has been shown that in human gastric biopsies of GML patients, a chemokine related to BAFF, called APRIL (A Proliferation Inducing Ligand) or TNFSF 13a, was produced by the infiltrating macrophages adjacent to neoplastic B cells [41]. APRIL is involved in the induction and maintenance of B and T cell responses, as well as in the promotion and survival of neoplastic cells [42]. It could therefore be interesting to investigate APRIL further, using our model, as this chemokine was absent of our panels of PCR and cytokine antibody arrays.

Autoimmune processes may be important in gastric lymphomagenesis. Indeed, BAFF is also known to affect the onset and severity of several autoimmune diseases [43]. Excessive production of BAFF helps the survival of low affinity and self-reactive B cells leading to a collapse of B cell tolerance [44]. Increased levels of this factor have been observed in the sera of patients with different types of autoimmune diseases, as well as in lymphoproliferative B cell diseases [45] [43]. Therefore the upregulation of BAFF in our GML mouse model may suggest involvement of an autoimmune attribute in this disease. The dysregulation of CD40 signalling has also been observed in multiple autoimmune diseases. The inhibition of LT and CD40 pathways has been shown to be effective in quieting inflammation in settings where DC-T cell interaction are key players of disease progression such as GML [46] [47].

In conclusion, the present study of the inflammatory response in mice developing GML following *H. pylori* infection allowed us to highlight several driving forces in this pathology: 1) the induction of a mixed inflammatory response, probably in line with the chemokine environment found at the lymphoma stage; 2) the upregulation of B cell promoting factors that have key functions in lymphoid organogenesis and 3) signs of a possible involvement of autoimmune phenomena. Our study also paves the way for future work on genetic predisposition for the dysregulation of some of TNF-superfamily members as putative genetic markers for this rare disease.

## MATERIALS AND METHODS

### RNA extraction

RNA was extracted from gastric biopsies using DNA/RNA/miRNA Universal Qiagen kit (Qiagen, Courtaboeuf, France): 50 and 40 RNA samples extracted from NTx (10 NI and 40 infected mice) and d3Tx mice (8 NI and 32 infected mice) respectively.

### Chemokines and cytokines PCR Array

The expression of chemokines and cytokines in the gastric mucosa of NTx and d3Tx mice was evaluated by PCR array using the commercialised “Mouse Cytokines & Chemokines panel” PAMM-150ZA-2 (Qiagen). For each model, a specific subset of 3 NI and 8 infected mice were investigated (reported in Chrisment *et al.* [11]). For d3Tx infected mice, animals that exhibited typical GML lesions were included, *i.e.* mice with B lymphoid infiltrates associated with lymphoepithelial lesions [11]. Moreover, only RNAs of high integrity and appropriate concentration after analysis on the Agilent 2100 Bioanalyzer (Agilent Technologies, Santa Clara, CA, USA) were considered: the final selection included 8 *H. pylori* infected d3Tx mice and 8 infected NTx mice.

500 ng of RNA was reverse transcribed to single-stranded cDNA using the RT^2^ First Strand kit (Qiagen) according to the manufacturer's protocol. Analysis of the expression of 84 inflammation mediator genes as well as that of five housekeeping genes (HKG) using the RT² SYBR Green Mastermix (Qiagen) was performed according to manufacturer's recommendations. PCR arrays were performed using the Stratagene MX3005p System (Agilent Technologies). Cycle thresholds were downloaded from the machine and analysed using the ΔΔCt method via Qiagen PCR array online system (http://www.sabiosciences.com/pcrarraydataanalysis.php). Data was normalised to the mean values of the five HKG. Fold-regulation calculations were performed using SABiosciences’ data analysis softwares. Fold-regulation values greater or less than two indicate an upregulation or downregulation, respectively.

### Quantitative real-time (RT)-PCR

Study of various genes’ expression in d3Tx and NTx mice stomachs was carried out by qRT-PCR.

Gastric RNA sample reverse transcription: the Primescript^TM^ RT Reagent Kit with gDNA Eraser (Perfect Real Time) (Takara, Saint-Germain-en-Laye, France) was used for reverse transcription of all purified RNA samples (750 ng) Primers used: among targets previously identified as overexpressed by PCR array, IL-4, IFNγ, LTα and LTβ were selected. Commercial primers targeting IL-4, LTα and LTβ (PPM03013F, PPM03114A and PPM03119A, respectively) and HKG Gusb (PPM05490C) (RT^2^ qPCR Primer Assay, Qiagen) were used. Primers targeting IFNγ and HKG hypoxanthine guanine phosphoribosyltransferase (*HPRT*), described by B. Flahou *et al.* [48] were synthesised by Eurofins MWG Biotech (Courtaboeuf, France). All of these primers allowed an evaluation of the relative expression levels for each target compared to HKG in stomachs. The primers were used at a concentration of 10 μM.

Amplification conditions: SYBR^®^ Premix Ex Taq^TM^ (Tli RNaseH Plus) (Takara) Mix was used for all qRT-PCRs. PCR experiments to amplify IL-4, IFNγ, LTα and LTβ and HKG *Gusb* and *HPRT* were performed in 384-well plates (Bio-Rad, Marnes la Coquette, France) with 3 μl of diluted cDNA (1/10) in a total volume of 10 μl. Each target was tested in triplicate for each sample. PCRs were carried out using a RT-PCR thermocycler CFX384^TM^ (Bio-Rad) at the TBM-Core real-time PCR platform (University of Bordeaux).

PCRs started with a 95°C DNA denaturation step for 3 minutes, followed by 40 cycles comprising 2 steps: a denaturation at 95°C for 5 seconds and a primer hybridisation at 60°C for 30 seconds. After each cycle, fluorescence was measured in order to quantify newly synthesised DNA. At the end of the procedure, a melting curve was generated by a slow elevation in the temperature from 65 to 95°C and the continuous measurement of fluorescence. The generation of this melting curve permitted the verification of one specific peak at the expected melting temperature for each product, which showed the PCR specificity.

Relative quantification of the targets’ expression: Cycle threshold (Ct) values above 35 were considered as non-specific and therefore not considered for the analysis. For each sample, Ct values obtained for each gene of interest were normalised in relation to the average of Ct values obtained for each HKG (ΔCt = Ct_gene of interest_ - Ct_housekeeping gene)._ The 2^−ΔCt^ value was then calculated, and enabled the results to be expressed as relative expression levels of genes of interest.

Relative expression levels of IL-4, IFNγ and LTα and LTβ genes for NI mice (control groups) were rationalised to 1, in order to normalise the expression levels in groups of infected mice in comparison to the expression levels in corresponding NI controls.

Relative expression levels of each target gene for the d3Tx mice group were also correlated with the histological scoring previously obtained [11].

### Cytokine antibody arrays

The expression of chemokines and cytokines in the gastric mucosa of d3Tx mice was evaluated at the protein level by cytokine antibody arrays (RayBio C-series, Mouse Cytokine Antibody Array C2000, RayBiotech, Inc, Norcross, GA, USA) according to the manufacturer's instructions. Five NI and 6 d3Tx infected mice which exhibited typical GML lesions were included [11].

Briefly, tissues were lysed in the cell lysis buffer provided after addition of proteinase inhibitors (Complete Mini, Roche, Basel, Switzerland), homogenized, glass-bead smashed (TissueLyser II, Qiagen) and centrifuged at 10,000 g for 10 min (4°C). The supernatants were recovered.

Protein extracts of each group of mice were pooled and pooled extracts diluted at least 10-fold in 1x blocking buffer to a total volume of 1.2 mL. After a blocking step, array membranes were incubated in pooled extracts at 4°C overnight. Each membrane was processed according to manufacturer's recommendations, incubated in ECL-plus and imaged on ImageQuant LAS 4000 (GE Healthcare Life Sciences, Velizy-Villacoublay, France).

Relative chemiluminescence intensity of each spot, corresponding to each protein, was recorded using ImageJ software. Data obtained with this software were analysed with RayBio Mouse Cytokine Antibody Array C Series 2000 Analysis Tool according to the manufacturer's instructions. Ratios of infected/NI d3Tx were calculated for each target and variation in expression was considered only when the ratio was > or equal to 2-fold.

### ELISA

The concentrations of BAFF were measured in the same protein extracts as above, by using a mouse BAFF/BLyS/TNFSF13B Immunoassay ELISA kit in accordance with the manufacturer's protocols (R&D systems, Minneapolis, MN). Plates were read using the Spectrostar Nano reader (BMG Labtech, Champigny sur Marne, France). All samples were tested in duplicate. Results were expressed in pg/mL.

### Statistical analysis

Statistical analyses were performed with GraphPad Prism 5.01 (GraphPad Software, Inc. La Jolla, CA, USA). Means were compared by the non-parametric Mann-Whitney test. Differences were considered significant when p was inferior to 0.05 (*p < 0.05).

## SUPPLEMENTARY MATERIAL TABLE


