# Supplementary material for: Characterisation of inflammatory processes in Helicobacter pylori-induced gastric lymphomagenesis in a mouse model
Source: Oncotarget. 2015 Oct 1;6(33):34525–36. doi: 10.18632/oncotarget.5948 (PMC4741470; doi:10.18632/oncotarget.5948)
Supplement: Supplementary file 1 [file oncotarget-06-34525-s001.pdf]

Characterisation of inflammatory processes in *Helicobacter pylori*-induced gastric lymphomagenesis in a mouse model

Supplementary Material

Supplemental Table 1. Upregulated targets identified by protein array in infected thymectomised mice.

	Target	Infected *vs* non infected d3Tx protein expression	PCR array upregulation
**Chemokines**	CXCL16	14.3	No
	CCL27	8.3	*nd*
	CXCL10	7.0	Yes
	CCL5	5.6	Yes
	CCL11	5.2	No
	CXCL5	3.2	No
	CXCL1	2.9	No
	CCL20	2.4	Yes
	CXCL13	2.3	Yes
	CCL19	2.2	No
	CCL1	2.0	Yes
	CCL3	2.0	Yes
	CCL17	2.0	No
	CCL25	2.0	*nd*
	CXCL2	2.0	*nd*
	CXCL12	2.0	No
**Th1 cytokines**	IL-2	4.1	No
	IL-12B	3.3	Yes
	IFN-γ	2.0	Yes
	IL-2 Rα	2.0	*nd*
	IL-12A	2.0	No
**Th2 cytokines**	IL-6 R	3.6	*nd*
	IL-13	2.1	Yes

**Th17 cytokines**	IL-21	2.5	No
	IL-17	2.3	No
**Lymphocyte activation and regulation**	CD40 L	26.5	Yes
	CD27 L	13.3	*nd*
	GITR L	6.8	*nd*
	IL-3 Rb	5.8	*nd*
	IL-3	4.8	No
	CD40	4.7	*nd*
	Growth arrest specific 6	4.4	*nd*
	CD30/TNFRSF8	3.3	*nd*
	CD27	3.2	*nd*
	Fcg RIIB	3.2	*nd*
	CD30L	3.0	*nd*
	IL-28	3.0	*nd*
	TNF-alpha	3.0	Yes
	IL1-alpha	2.9	Yes
	IL1-beta	2.9	Yes
	GITR	2.8	*nd*
	sTNF RII	2.8	*nd*
	IL-20	2.3	*nd*
	TWEAK R	2.3	*nd*
	sTNF RI	2.2	*nd*
	TWEAK	2.2	*nd*
	TACI	2.0	*nd*
**Others**	Granzyme B	8.1	*nd*
	JAM-A	6.1	*nd*
	L-Selectin	5.5	*nd*
	CD36	4.2	*nd*
	VCAM-1	4.1	*nd*
	SCF	3.4	*nd*
	Flt-3 Ligand	2.7	*nd*
	P-Selectin	2.7	*nd*
	Leptin R	2.6	*nd*
	IGF-BP-6	2.4	*nd*

TPO	2.3	*nd*
VEGF	2.3	No
PF4	2.2	*nd*
TIMP-1	2.2	*nd*
axl	2.0	*nd*
GM-CSF	2.0	No
IGF-BP-5	2.0	*nd*
TREM-1	2.0	*nd*

(fold-regulation $\geq$2)

nd: not determined; L=ligand.
